# Development of an HPTLC-MS Method for the Differentiation of Celosiae Semen: *Celosia argentea* Versus *C. cristata*

**DOI:** 10.3390/molecules30132786

**Published:** 2025-06-28

**Authors:** Kyu Won Kim, Geonha Park, Sejin Ku, Young Pyo Jang

**Affiliations:** 1 Department of Biomedical and Pharmaceutical Sciences, Graduate School, Kyung Hee University, Seoul 02447, Republic of Korea; rbdnjs1127@naver.com (K.W.K.); zbxl0910@naver.com (S.K.); 2 Division of Pharmacognosy, College of Pharmacy, Kyung Hee University, Seoul 02447, Republic of Korea; ginapark0326@khu.ac.kr; 3 Department of Oriental Pharmaceutical Sciences, College of Pharmacy, Kyung Hee University, Seoul 02447, Republic of Korea; 4 Department of Integrated Drug Development and Natural Products, Graduate School, Kyung Hee University, Seoul 02447, Republic of Korea

**Keywords:** Celosiae Argentea Semen (CAS), *Celosia argentea* L., *Celosia cristata* L., classification, high-performance thin-layer chromatography—mass spectrometry (HPTLC–MS)

## Abstract

Celosiae Argentea Semen (CAS), derived from *Celosia argentea* L., is traditionally used in Korean and Chinese medicine to treat eye disorders and liver heat and is recognized in official Pharmacopeias. In contrast, Celosiae Cristatae Semen (CCS), despite its frequent presence in the market, is not officially listed. The morphological and chemical similarities between the two pose challenges for accurate identification. This study presents an integrative method combining digital image analysis and high-performance thin-layer chromatography coupled with mass spectrometry (HPTLC-MS) to differentiate CAS from CCS. Digital microscopy and ImageJ analysis showed that CCS has a projection area over twice that of CAS. Chemically, an optimized HPTLC method using ethyl acetate, methanol, water, and formic acid revealed distinct fingerprint patterns under UV 366 nm and white light. Notably, celosin F was exclusively detected in CAS, while celosin H, J, and K were characteristic of CCS. ESI-TOF-MS analysis confirmed these markers, resolving an overlap in *R*_F_ values. Repeatability tests showed total SDs of sucrose for intra-day, inter-day, and inter-analysis precision were 0.006, 0.004, and 0.005, respectively, confirming method reliability. This combined approach offers a rapid, reliable, and practical tool for distinguishing these two medicinal seeds, supporting enhanced quality control and regulatory standardization in pharmaceutical applications.

## 1. Introduction

Celosiae Argentea Semen (CAS) has been traditionally used in China (known as Qingxiangzi) and Korea (known as Cheong Sang Ja). In traditional medicine, CAS is primarily used to treat eye disorders caused by liver heat and is also known to be effective in alleviating high blood pressure and dizziness associated with liver heat [1,2]. The primary active components of CAS are triterpenoid saponins, including celosin A to M. Among them, celosin A to D, I, II, K and L exhibit hepatoprotective and antitumor effects by preventing the elevation of hepatic enzymes [3,4,5,6]. Celosin E, F, and G are recognized for their anti-inflammatory and antitumor [7], whereas celosin H to J and M are recognized for their neuroprotective activities [8,9]. Additionally, bicyclic peptides such as celogentin possess antimitotic activity by inhibiting tubulin polymerization [10,11]. In addition to these bioactive constituents, the water extract of CAS is well known for its antioxidant properties, which may contribute to the prevention of eye damage [12]. Also, the 50% alcoholic extract of CAS exhibited anti-diabetic activity in alloxan-induced diabetic rats [13].

According to the Korean Herbal Pharmacopoeia (KHP) and Pharmacopoeia of the People’s Republic of China (ChP 2020), CAS is listed as the seed of *Celosia argentea* L. as its sole origin, while *C. cristata* L. is not listed as an official medicinal herb. Nonetheless, due to their morphological and chemical similarities, Celosiae Cristatae Semen (CCS) is often marketed as CAS—a practice that raises significant concerns. Given these morphological and chemical parallels, research on CCS has focused on identifying its components and revealing its structural characteristics including key compounds such as celosin A to D and H to M that closely mirror that of CAS [14,15,16,17,18]. Even though these two species show similar bioactivities derived from common compounds, CAS contains unique components, such as celosin E to G and celogentins, which are absent in CCS and contribute to differences in efficacy. Therefore, distinguishing CAS from CCS is essential, particularly when considering the anticancer applications of CAS [7,10,11], to ensure both quality control and therapeutic effectiveness. However, research establishing clear criteria to differentiate CAS from CCS remains insufficient. Additionally, some studies suggest that *C. argentea* is the direct progenitor of *C. cristata* as *C. argentea* var. *cristata* [19,20]. These similarities make differentiation challenging, further emphasizing the need for clear distinction and separate usage of CAS and CCS.

Recent studies have explored various approaches to differentiate CAS from CCS, including Sequence-Related Amplified Polymorphism (SRAP), High-Performance Liquid Chromatography (HPLC) fingerprinting, and flower morphology [20,21,22]. In the SRAP method, 22 *Celosia* populations were genetically divided into two distinct clades—*C. argentea* (Cluster I) and *C. cristata* (Cluster II)—with a specific fragment, M1E6, identified as a discriminant marker [20]. In the HPLC method, the peak area ratio of celosin H to celosin I was proposed as a discriminant marker, offering a chemical basis for distinguishing between the two species. Additionally, advanced microscopic techniques such as stereomicroscopy, SEM, and polarized light microscopy were employed to establish detailed morphological diagnostic keys distinguishing between the two species [21]. Morphological differences in the flowers also provide an additional marker of discrimination [22].

In this study, we adopted a simplified yet effective approach using ImageJ software to identify distinguishing morphological features based on the two-dimensional vertical projection area of each seed. A total of 14 Celosiae Semen samples were measured and classified into two groups according to size. Statistical analysis confirmed a significant difference between the groups, supporting the morphological differentiation between CAS and CCS. Compared to high-cost microscopic instrumentation, this method provides a practical and accessible alternative for rapid screening, particularly in resource-limited environments.

In addition to morphological analysis, this study highlights the practical utility of High-Performance Thin-Layer Chromatography (HPTLC) as a rapid, cost-effective, and accessible tool for differentiating CAS from CCS. When combined with visualization reagents, HPTLC produced clear and reproducible separation patterns, enabling effective visual comparison between the fingerprints of the two species. Distinct chemical fingerprints were observed, and characteristic bands were identified as markers for species-level discrimination. These bands were further analyzed using Electrospray Ionization–Time of Flight Mass Spectrometry (ESI–TOF–MS) for identification and annotation compounds. This led to the establishment of a rapid and accurate technique for distinguishing CAS from CCS. Additionally, this HPTLC-MS approach enabled straightforward visual confirmation of differences in celosin-series compounds between the two species, providing a practical and efficient tool for seed identification in pharmaceutical markets.

## 2. Results

This study established a comprehensive approach combining morphological assessment with an optical digital microscope and TLC-based fingerprint analysis employing HPTLC-MS to effectively differentiate CAS from CCS. While visual inspection alone is insufficient for reliable differentiation between the two species, the use of an optical digital microscope in conjunction with ImageJ clearly confirms differences in seed sizes. Furthermore, as existing Pharmacopeial identification tests are not tailored to distinguish CAS from CCS, this research offers optimized HPTLC-MS methods to identify distinct bands, enabling efficient differentiation and identification of CAS and CCS.

### 2.1. Size Assessment via ImageJ

**Figure 1:**
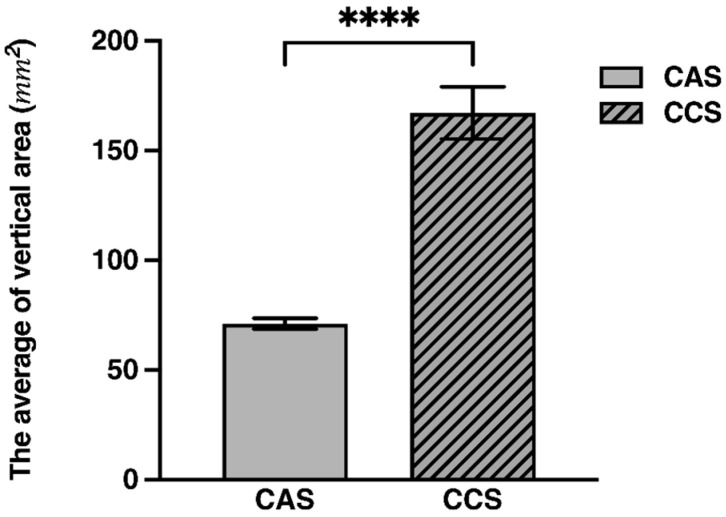
Comparison of mean vertical projection areas of CAS (71.12 mm^2^) and CCS (167.18 mm^2^) (*p* < 0.0001), indicating a highly significant difference. Asterisks denote statistical significance; **** represents *p* < 0.0001.

CAS and CCS are challenging to distinguish visually due to their similar color, shape, and texture. However, CCS has been reported to have a slightly glossier surface and a larger seed size compared to CAS [23]. Building on these observations, this study employed a digital imaging approach using standardized photographs taken under controlled conditions to enhance precision in morphological evaluation. ImageJ software was then utilized to calculate the two-dimensional vertical projection area of each seed, providing a reproducible dataset for comparison. The samples were grouped into CAS (*n* = 7) and CCS (*n* = 7), and the projection area of each sample was measured ten times to obtain an average value (Table S1). The resulting average projection areas for each group were compared and visualized using a grouped bar graph (Figure 1), with error bars representing the standard deviation (SD). Due to differences in variance between the groups, a Mann-Whitney U test was conducted to assess whether the distribution of projection areas differed significantly between the CAS and CCS groups. The test results (U statistic = 0.00, Z-score=−10.2085, *p* < 0.000001) indicated a statistically significant difference, with the median projection area of CCS being more than twice that of CAS (Table 1).

The morphologically classified samples were subsequently analyzed by HPTLC, and the results showed strong agreement with the ImageJ-based classification, further supporting the validity of the species grouping.

### 2.2. HPTLC Fingerprints of CAS and CCS

To evaluate the consistency across individual HPTLC plates, a system suitability test (SST) was performed using the Universal HPTLC Mix (UHM). Under the prescribed HPTLC parameters, the UHM chromatogram was clearly detected under UV 254 nm. Among the observed bands, the three most intense were selected as reference markers in descending order of their *R*_F_ values. Based on nine replicate plates, SST acceptance criteria were established as 0.844 ± 0.010, 0.792 ± 0.010, and 0.668 ± 0.015 for selected markers (Table S2). Using these criteria, the system suitability of plate 1 and 2, which are used for the analysis of samples and standard mixtures, was evaluated and confirmed to be acceptable (Figure S1).

To further assess method performance, repeatability and stability tests were conducted. In the repeatability assessment, intra-day, inter-day, and inter-analyst precision were evaluated using the *R*_F_ values of sucrose, celosin J, celosin I, and celosin H. Among these, sucrose’s standard deviations (SDs) were 0.006, 0.004, and 0.005 for intra-day, inter-day, and inter-analyst precision, respectively, indicating high repeatability across all conditions (Figures S5–S7 and Tables S3–S5). Stability of the sample solution and photographing time was also investigated. After 8 h, the color intensity of the sucrose band under white light slightly faded, but its *R*_F_ value remained sufficiently visible for accurate *R*_F_ determination (Figure S8). The overall chromatographic profile was well maintained within 60 min after derivatization, confirming the stability of the plates during the photographing period (Figure S9). Robustness was evaluated with respect to developing distance. When the developing distance varied from 68 mm to 85 mm, the SD of the *R*_F_ values for all four standards remained below 0.006, demonstrating acceptable robustness of the method (Figure S10). Although the present method was developed for qualitative fingerprinting purposes, future work including standard curve generation and sensitivity evaluations will be necessary to define detection limits and enable quantitative application. 

**Figure 2:**
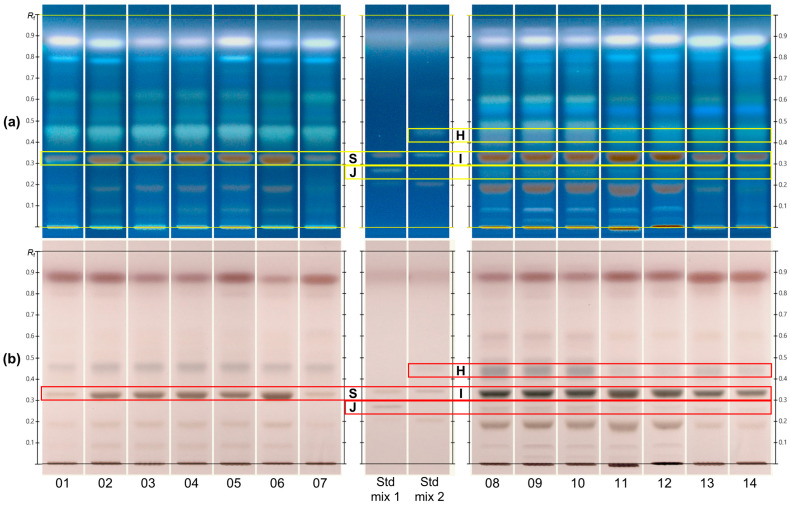
HPTLC fingerprints of CAS (track 01 to 07) and CCS (track 08 to 14) samples under UV (**a**) 366 nm; and (**b**) White light after derivatization using a 10% sulfuric acid reagent. Track 01 to 05 and 08 to 14: Plate 1; Track 06, 07, 15, and 16: Plate 2; Std mix 1: celosin J (J) and sucrose (S); Std mix 2: celosin I (I) and celosin H (H).

An optimized solvent system consisting of ethyl acetate, methanol, water, and formic acid (13:7:1:1, *v*/*v*/*v*/*v*) was developed for HPTLC analysis. Following derivatization with 10% sulfuric acid in methanol, distinct spots were observed under UV light at 366 nm and white light (Figure 2). Despite overall similarity in the HPTLC chromatograms of CAS and CCS, the samples consistently clustered into two distinct HPTLC fingerprints, which were independent of the supplier. 

The CAS samples exhibited major spots at *R*_F_ values of 0.33, 0.46, and 0.62 detected exclusively in CAS. Conversely, CCS displayed distinct bands at *R*_F_ 0.25, 0.33, 0.44, and 0.60. Sucrose (*R*_F_ 0.32) was present in both CAS and CCS, whereas celosin H (*R*_F_ 0.44), celosin I (*R*_F_ 0.33), and celosin J (*R*_F_ 0.25) were detected exclusively in CCS. To identify these spots, sucrose and celosin H to J were compared with available reference standards. The original HPTLC plate images used for this comparison are presented in Figures S2–S4. Specifically, plate 0 contains the individual standard solutions to confirm the *R*_F_ values of sucrose and celosins, while plate 1 and 2 show the chromatographic profiles of the samples and standard mixtures. Notably, the *R*_F_ values of sucrose and celosin I overlapped, complicating their differentiation based solely on HPTLC data. To resolve this, mass spectrometric (MS) analysis was employed, providing accurate identification and additional annotation by matching the mass spectra with the known literature data. 

### 2.3. Advanced Chemical Profiling of HPTLC-Derived Spots by MS-Interface

HPTLC-MS analysis was performed to identify distinct spots observed in the HPTLC fingerprints of CAS and CCS by comparing their mass–to–charge ratios (*m*/*z*) and calculated molecular formulas against the literature data [21,24]. One representative sample from each group (CAS 5 and CCS 8) was selected for MS analysis based on the highest band intensity. Each selected sample was analyzed in triplicate, and a representative spectrum was selected based on clarity and reproducibility of the peaks. In addition, bands selected for MS analysis were prioritized based on their intensity, resolution, and relevance to distinguishing CAS from CCS. This approach led to the detection of multiple triterpenoid saponins and a disaccharide, including celosin F, celosin K, celosin H, celosin I, celosin J, and sucrose.

**Figure 3:**
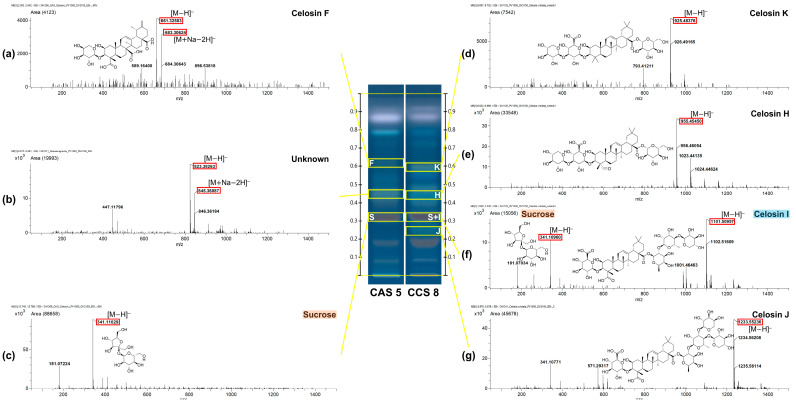
Representative HPTLC fingerprints and mass spectra of the major spots. (**a**) celosin F in CAS; (**b**) undefined compound in CAS; (**c**) sucrose in CAS; (**d**) celosin K in CCS; (**e**) celosin H in CCS; (**f**) sucrose and celosin I in CCS; (**g**) celosin J in CCS.

As summarized in Figure 3 and Table 2, bands are labeled by lowercase letters (a) to (g) according to their *R*_F_ positions, and capital letters indicate the corresponding compounds (e.g., H for celosin H, S for sucrose), including both identified and annotated compounds based on MS features. Compound a (*R*_F_ 0.62) exhibited molecular ion peaks at *m*/*z* 661.32940 [M−H]^−^ and *m*/*z* 683.30624 [M+Na−2H]^−^ consistent with C_35_H_50_O_12_ and annotated as celosin F (F). Compound b (*R*_F_ 0.46) exhibited molecular ion peaks at *m*/*z* 823.38263 [M−H]^−^ and *m*/*z* 845.36087 [M+Na−2H]^−^, from which the molecular formula C_34_H_64_O_22_ was inferred, but the compound remains undefined. Compound c (*R*_F_ 0.33) corresponded to sucrose (S), showing a [M−H]^−^ ion at *m*/*z* 341.11029, consistent with the molecular formula C_12_H_22_O_11_. In the CCS fingerprint, compound d (*R*_F_ 0.60) was annotated as celosin K (K), presenting a [M−OH]^−^ ion at *m*/*z* 925.48376 corresponding to C_47_H_74_O_19_. Compound e (*R*_F_ 0.44) was defined as celosin H (H), showing a [M−H]^−^ ion at *m*/*z* 955.4540, consistent with C_47_H_72_O_20_. Compound f (*R*_F_ 0.33) represented a co-elution of sucrose (S), with a [M−H]^−^ ion at *m*/*z* 341.10960, and celosin I (I), with a [M−H]^−^ ion at *m*/*z* 1101.50957 corresponding to C_53_H_82_O_24_. Finally, compound g (*R*_F_ 0.25) was identified as celosin J (J), displaying a [M−H]^−^ ion at *m*/*z* 1233.55236 and the molecular formula C_58_H_90_O_28_ (Table 2).

All compounds were annotated based on the literature MS data or MassBank. In contrast, sucrose, celosin H, celosin I, and celosin J were identified through direct comparison with authentic standards with the exception of compound celosin F and celosin K. Additionally, a consistent band at *R*_F_ 0.20 was detected in both CAS and CCS, as well as in celosin-type standards (celosin H, I, and J). In negative ion mode, this band exhibited a dominant ion at *m*/*z* 503.16328, tentatively corresponding to C_18_H_32_O_16_ [M−H]^−^ or a possible chlorinated fragment of celosin-type compounds (e.g., C_30_H_28_ClO_5_). While the precise structure remains unresolved, its presence in multiple celosin standards suggests that it may represent a common fragment or degradation product associated with celosin-type triterpenoid glycosides (Figure S11). Furthermore, the light purple fluorescent band observed at *R*_F_ value of 0.85 under UV 366 nm exhibited a molecular ion peak [M+K]^+^ at *m*/*z* 319.20394 and was annotated as linoleic acid under positive ion mode (Figure S11).

## 3. Discussion

Interestingly, in the CAS fingerprint, a spot with an *R*_F_ value similar to celosin H (compound b) produced a molecular ion peak at *m*/*z* 823.38253 corresponding to C_34_H_64_O_22_, a formula that does not match any currently reported celosin-type compounds, including celosins or celogentins, to the best of our knowledge. While this may suggest the presence of an uncharacterized celosin-related compound, this tentative assignment is based solely on accurate mass measurement. Further structural elucidation using MS/MS or NMR would be required to confirm its identity and assess its novelty. Nevertheless, the consistent detection of this molecular ion indicates a promising candidate for future study. 

In addition, while sucrose was detected in both CAS and CCS at *R*_F_ 0.33, the CCS sample at the same *R*_F_ revealed an additional ion peak corresponding to celosin I. Notably, these findings differ from previous reports indicating that celosin H, I, J, and K occur in both CAS and CCS [9,16], whereas celosin F is exclusive to CAS. This discrepancy underscores how HPTLC’s lower sensitivity, compared with techniques such as (U)HPLC or MS, can lead to concentration-dependent variability in compound detection. At the same time, it also highlights HPTLC’s unique capacity for TLC-specific differentiation.

Although the current study successfully established a proof-of-concept using 14 market batches from two regions, this sampling remains geographically limited. For future pharmacopeial adoption and broader regulatory acceptance, additional studies incorporating wider geographic representation will be essential to confirm the consistency and generalizability of these findings.

Consequently, TLC-based methods offer clear distinguishing features between CAS and CCS. Moreover, their distinct chemical compositions likely contribute to variations in bioactivity, implying that CAS and CCS should be used separately in medicinal applications to optimize therapeutic efficacy.

## 4. Materials and Methods

### 4.1. Plant Materials and Sample Preparation 

**Figure 4:**
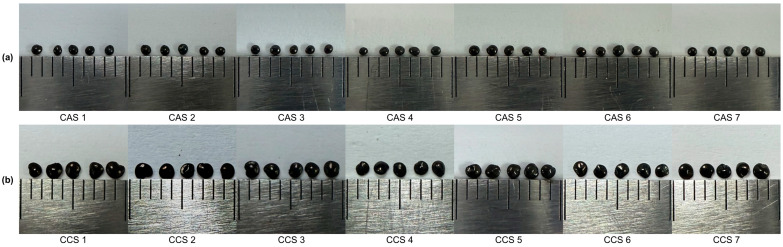
Morphological images of the 14 Celosiae Semen samples (**a**) CAS; (**b**) CCS.

A total of 14 batches of Celosiae Semen, consisting of 7 batches of CAS and 7 batches of CCS, were procured from the local market (Table 3). Morphological images of all 14 samples are shown in Figure 4. The morphological classification of Celosiae Semen was conducted prior to chemical analysis based on traditional diagnostic features such as seed shape, surface texture, and color. This classification followed previously established criteria for fine seed herbs using stereoscopic observation methods [23].The authenticity of the collected samples was organoleptically confirmed by Prof. Young Pyo Jang from Kyung Hee University in South Korea.

Each sample was ground into a fine powder and passed through an 850 μm sieve. Subsequently, 1.0 g of the sieved powder was mixed with 10 mL of 50% ethanol and decocted for 1 h in a water bath at 80 °C. The supernatant was then filtered off, and the residue was subjected to the same extraction procedure a second time. The combined filtrates were evaporated, and the resulting residue was dissolved in 1 mL of 50% ethanol. Finally, the solution was filtered through a 0.45 μm Polyvinylidene fluoride (PVDF) syringe filter (Whatman, Marlborough, MA, USA) to obtain the test solution.

### 4.2. Chemicals and Reagents 

Methanol (99.8%), ethanol (94.0%), ethyl acetate (99.5%), and magnesium chloride hexahydrate (98.0%) were obtained from Duksan (Ansan, Republic of Korea), formic acid (99.0%) from Daejung Chemicals & Metals (Siheung, Republic of Korea), sulfuric acid (95.0%) from Samchun Pure Chemical Co., Ltd. (Pyeongtaek, Republic of Korea). Water (HPLC grade), and methanol (HPLC grade) were obtained from ThermoFisher Scientific (Waltham, MA, USA). Standard compounds, celosin H (98.0%, CFN91669-CFS202301), celosin I (99.9%, CFN91061-CFS202201), celosin J (98.0%, CFN91152-CFS202402) were supplied by Chemfaces (Wuhan, China), sucrose (99.5%, S9378-102453507) from Sigma-Aldrich (St. Louis, MO, USA). Individual standards were dissolved in 50% ethanol to create solution stocks with a concentration of 1.0 mg/mL.

### 4.3. ImageJ Analysis

For the size comparison of CAS and CCS, 10 seeds were randomly selected from each batch. Each seed was placed on a flat surface, and a camera was positioned vertically overhead to capture top-view images. The images were then processed using ImageJ software (version 1.54g, National Institutes of Health, Bethesda, MD, USA) [25], which converts pixel intensity data into binary form to facilitate precise measurement of the two-dimensional projection area. After converting an image to 8-bit grayscale, the threshold values were set with 0 as the minimum and 70 as the maximum. A reference scale present in the image was used for calibration, ensuring accurate area measurements in physical units (e.g., mm^2^). All measured data were subsequently exported as CSV files for further analysis. Statistical analysis was then performed using Statistica (version 13.5.0.17), and the Mann-Whitney U test was employed to determine whether the distribution differences between the two groups were statistically significant.

### 4.4. HPTLC-MS Analysis 

#### 4.4.1. Equipment

HPTLC examinations were conducted using 20 × 10 cm silica gel 60 F_254_ glass plates (Merck, Darmstadt, Germany) across several HPTLC setups provided by CAMAG (Muttenz, Switzerland). These setups comprised a Linomat 5 semiauto-applicator, an Automatic Developing Chamber (ADC 2), a Derivatizer, a Visualizer 2, and visionCATS software, version 3.1. visionCATS software facilitated data analysis, system control, and quantification. For sample application, a 100 µL syringe (Hamilton, Bonaduz, Switzerland) was used in conjunction with nitrogen gas (Sinyang Sanso, Seoul, Republic of Korea).

#### 4.4.2. HPTLC Conditions

For HPTLC, a total of 14 sample solutions were applied in volumes of 2 µL, forming 8 mm bands with a spacing of 11.4 mm and positioned 10 mm from the plate’s bottom edge, at a dosage speed of 50 nL/s on 20 × 10 cm plates. The initial band was placed 10 mm from the plate’s side edge. The development process was standardized by conditioning the layer at 33% relative humidity using a saturated aqueous solution of magnesium chloride for 10 min. After conditioning, the plates were developed in a chamber pre-saturated for 20 min with a saturation pad, allowing the solvent front to reach a distance of 78 mm from the plate’s bottom edge. The plates were then dried for 5 min. The developing solvent consisted of ethyl acetate, methanol, water, and formic acid, mixed in a volumetric ratio of 13:7:1:1, yielding a total volume of 44 mL. Of this mixture, 10 mL was used for the development process, while 25 mL was allocated for saturating the chamber. After development, derivatization was carried out using a 10% sulfuric acid reagent, prepared by dissolving 10 mL of concentrated sulfuric acid in 90 mL of methanol under cooling, as per the CAMAG-recommended method. The derivatized plate was subsequently heated on a hot plate at 100 °C for 3 min. HPTLC chromatograms were finally captured under UV light at 366 nm and white light for profiling.

For a system suitability test (SST), the Universial HPTLC mix (UHM) [26,27], which consists of guanosine, sulisobenzone, thymidine, paracetamol, phthalimide, 9-hydroxyfuorene, thioxanthen-9-one and 2-(2H-benzotriazol-2-yl)- 4-(1,1,3,3 tetramethylbutyl)-phenol, was used (CAMAG, Muttenz, Switzerland).

#### 4.4.3. MS-Interface Conditions

For the identification of compounds corresponding to specific spots on the HPTLC plate, mass spectrometry data were obtained using a JMS-T100TD Time of Flight (TOF) mass spectrometer (JEOL Ltd., Tokyo, Japan) equipped with an electrospray ionization (ESI) source. The CAMAG TLC-MS interface, designed to integrate chromatographic and MS analyses, was connected to both a binary pump and the mass spectrometer. To precisely select chromatographic zones for HPTLC-MS analysis, these regions were marked under UV light at 366 nm. This step ensured accurate positioning of the HPTLC plate beneath the oval elution head (2 × 4 mm) of the TLC-MS interface, enabling efficient compound elution into the mass spectrometer. The elution process was performed at a flow rate of 0.3 mL/min, using 80% methanol as the eluent. The MS settings were configured as follows: a mass-to-charge ratio (*m*/*z*) range of 50 to 1500 with a scan interval of 0.5 s, and analysis was conducted in negative ion mode. Key parameters included an orifice 1 temperature of 80 °C, a desolvating chamber temperature of 250 °C, detector voltage set at 2100 V, ring lens voltage at −15 V, orifice 1 voltage at −80 V, orifice 2 voltage at −7 V, needle voltage at −2000 V, peak voltage at 1500 V, nebulizing nitrogen gas flow at 1 L/min, and desolvating nitrogen gas flow at 3 L/min. For accurate mass measurement, the mass scale was calibrated using the YOKUDELNA solution (JEOL Ltd.), and data acquisition was managed using Mass Center software, version 1.3.7b (JEOL Ltd.).

The levels of compound identifications was assigned following the Metabolomics Standards Initiative (MSI) guidelines [28]. Compounds confirmed with standards were classified as Level 1 (identified), whereas those matched to the literature MS data without standard comparison were considered Level 2 (annotated).

## 5. Conclusions

In conclusion, the integrative approach utilized in this study offers a practical and reliable strategy for differentiating CAS from CCS by leveraging both visual and chemical profiles. Although there are limitations in identifying individual compounds using HPTLC alone, the distinctive fingerprint patterns obtained are sufficient to distinguish between the two species. For instance, while one spot in the CAS fingerprint initially displayed an *R*_F_ value similar to celosin H, MS analysis confirmed that it was a different compound. Likewise, the overlapping *R*_F_ values of sucrose and celosin I in CCS complicated compound identification through HPTLC alone. Nonetheless, these challenges do not detract from the utility of HPTLC, especially when complemented by MS analysis, in effectively identifying key differences between CAS and CCS.

Ultimately, the findings presented here establish a solid foundation for the development of stricter regulatory guidelines and standardization efforts not only for Celosia species but also for other medicinal plants, thereby helping to ensure that their therapeutic potential is fully realized.

## Figures and Tables

**Table 1:** Results of the Mann–Whitney U test comparing the CAS and CCS groups, including Rank Sum, U statistic, Z-score, *p*-value, Z adjusted, and two-sided exact *p*.

Rank Sum		U Statistic	Z-Score	*p*-Value	Z Adjusted	Valid N		2*1 Sided Exact *p*
CAS	CCS					CAS	CCS	
2485	7385	0.0	−10.2085	0.000	−10.2085	70	70	0.000

**Table 2:** Identification of triterpenoid saponins in CAS and CCS by MS interface in negative ion mode.

No.	*R*_F_ Value	*m*/*z* ^a^	Quasi-Molecular Ion	Mass Difference (mmu)	Molecular Formula	Identification	Ref
(a)	0.62	661.32583	[M−H]^−^	3.43	C_35_H_50_O_12_	celosin F *	[21] ^c^
		683.30624	[M+Na−2H]^−^	1.89			
(b)	0.46	823.38263	[M−H]^−^	1.53	C_34_H_64_O_22_	Undefined	-
		845.36087	[M+Na−2H]^−^	−2.18			
(c)	0.33	341.11029	[M−H]^−^	1.90	C_12_H_22_O_11_	sucrose	[24] ^b,c^
(d)	0.6	925.48376	[M−OH]^−^	4.07	C_47_H_74_O_18_	celosin K	[21] ^c^
(e)	0.44	955.45450	[M−H]^−^	0.63	C_47_H_72_O_20_	celosin H	[21] ^b,c^
(f)	0.33	341.10960	[M−H]^−^	1.22	C_12_H_22_O_11_	sucrose	[24] ^b,c^
	0.33	1101.50957	[M−H]^−^	−2.20	C_53_H_82_O_24_	celosin I	[21] ^b,c^
(g)	0.25	1233.55236	[M−H]^−^	−1.68	C_58_H_90_O_28_	celosin J	[21] ^b,c^

^a^ Mass-to-charge ratio (*m*/*z*); ^b^ identified with standard compounds; ^c^ annotated by comparison with literature or MassBank; * Only present in CAS.

**Table 3:** Origin and summary of the 14 Celosiae Semen samples.

No.	Sample	Botanical Source	Origin	Supplier	Expiration Date
1	Celosiae Semen	*C. argentea* L.	China	Mageherb	30 June 2025
2	Celosiae Semen	*C. argentea* L.	China	Donguibogam	18 May 2024
3	Celosiae Semen	*C. argentea* L.	China	Hanteut Herbal Medicine	10 May 2025
4	Celosiae Semen	*C. argentea* L.	China	Hanteut Herbal Medicine	26 February 2025
5	Celosiae Semen	*C. argentea* L.	China	Hanteut Herbal Medicine	9 May 2024
6	Celosiae Semen	*C. argentea* L.	China	Dongui Hanjae	5 July 2024
7	Celosiae Semen	*C. argentea* L.	China	Allborn	30 June 2025
8	Celosiae Semen	*C. cristata* L.	China	Gyeongshin Seeds	30 January 2025
9	Celosiae Semen	*C. cristata* L.	China	Danong	30 March 2025
10	Celosiae Semen	*C. cristata* L.	China	Aram Seeds	30 January 2025
11	Celosiae Semen	*C. cristata* L.	China	DaeHyo Pharmaceutical	14 June 2025
12	Celosiae Semen	*C. cristata* L.	China	Gwangmyeongdang Pharmaceutical	24 May 2024
13	Celosiae Semen	*C. cristata* L.	China	Human Herb	12 January 2025
14	Celosiae Semen	*C. cristata* L.	China	DaeHyo Pharmaceutical	22 January 2023

## Data Availability

No new data were created or analyzed in this study. Data sharing is not applicable to this article.

## Supplementary Materials

The following supporting information can be downloaded at: https://www.mdpi.com/article/10.3390/molecules30132786/s1, Table S1: Comparison of mean vertical projection areas of CAS and CCS; Table S2: Summary of *R*_F_ values and variability for system suitability test (SST) acceptance criteria (n = 9); Table S3: *R*_F_ Values of Celosin J, Sucrose, Celosin I, and Celosin H in the intra-day precision of the Repeatability assessment. The identifiers (a), (b), and (c) correspond to the respective plates illustrated in Figure S5; Table S4: *R*_F_ Values of Celosin J, Sucrose, Celosin I, and Celosin H in the inter-day precision of the Repeatability assessment. The identifiers (a), (b), and (c) correspond to the respective plates illustrated in Figure S6; Table S5: *R*_F_ Values of Celosin J, Sucrose, Celosin I, and Celosin H in the inter-analyst precision of the Repeatability assessment. The identifiers (a), (b), and (c) correspond to the respective plates illustrated in Figure S7; Table S6: *R*_F_ Values of Celosin J, Sucrose, Celosin I, and Celosin H in the stability of sample solutions of the Stability assessment. The identifiers (a) and (b) correspond to the respective plates illustrated in Figure S8; Table S7: *R*_F_ Values of Celosin J, Sucrose, Celosin I, and Celosin H in the Robustness assessment using different developing distance. The identifiers (a), (b), and (c) correspond to the respective plates illustrated in Figure S10. Figure S1: System suitability evaluation of plate 1 (a) and 2 (b) based on acceptance criteria defined using the UHM. The red lines indicate the upper and lower thresholds; Figure S2: Original image of HPTLC plate 0 under UV (a) 366 nm with an exposure time of 5.034 s and (b) White light with an exposure time of 0.088 s. Track 01 to 03, and 13 to 15, blank; Track 04, 08, and 12, UHM (system suitability test reference); Track 05, CAS 5 (sample); Track 06, Sucrose (standard); Track 06, Celosin J (standard); Track 09, CCS 8 (sample); Tracks 10, Celosin H (standard); Track 11, Celosin I (standard). 20.0 °C and 38%; Figure S3: Original image of HPTLC plate 1 under UV (a) 366 nm with an exposure time of 7.165 s and (b) White light with an exposure time of 0.068 s. Track 01, 08, and 15, UHM (system suitability test reference); Track 02 to 06, CAS 1 to CAS 5 (samples); Track 07, Celosin I and Celosin H (standard mix, as increasing *R*_F_); Track 09, Celosin J and Sucrose (standard mix, as increasing *R*_F_); Track 10 to 14, CCS 8 to CCS 12 (samples). 21.0 °C and 39%; Figure S4: Original image of HPTLC plate 2 under UV (a) 366 nm with an exposure time of 5.729 s and (b) White light with an exposure time of 0.056 s. Track 01, 08, and 15, UHM (system suitability test reference); Track 02 to 04, CAS 3, CAS 2, and CAS 4 (samples); Track 05 and 06, CAS 6 and CAS 7 (samples); Track 07, Celosin J and Sucrose (standard mix, as increasing *R*_F_); Track 09, Celosin I and Celosin H (standard mix, as increasing *R*_F_); Track 10 to 14, CCS 10 to CCS 14 (samples). 21.0 °C and 39%; Figure S5: Repeatability assessment of the HPTLC method to evaluate intra-day precision based on three replicate analyses. Chromatograms obtained from three independently prepared sample solutions of the same material, standard mix, and UHM under UV 366 nm and White light. Track 01, CAS 5; Track 02, Celosin J and Sucrose (standard mix, as increasing *R*_F_); Track 03, UHM (system suitability test reference); Track 04, Celosin I and Celosin H (standard mix, as increasing *R*_F_); Track 05, CCS 8. (a), 21.0 °C and 39%; (b), 21.0 °C and 39%; (c), 22.0 °C and 39%; Figure S6: Repeatability assessment of the HPTLC method to evaluate inter-day precision based on analyses conducted on three separate days. Chromatograms obtained from three different sample solutions prepared on three days, standard mix, and UHM under UV 366 nm and White light. Track 01, CAS 5; Track 02, Celosin J and Sucrose (standard mix, as increasing *R*_F_); Track 03, UHM (system suitability test reference); Track 04, Celosin I and Celosin H (standard mix, as increasing *R*_F_); Track 05, CCS 8. (a), 22.0 °C and 39%; (b), 22.0 °C and 39%; (c), 20.0 °C and 39%; Figure S7: Repeatability assessment of the HPTLC method to evaluate inter-analyst precision based on analysis of different analysts. Chromatograms obtained from three sample solutions independently prepared by 3 operators, standard mix, and UHM under UV 366 nm and White light. Track 01, CAS 5; Track 02, Celosin J and Sucrose (standard mix, as increasing *R*_F_); Track 03, UHM (system suitability test reference); Track 04, Celosin I and Celosin H (standard mix, as increasing *R*_F_); Track 05, CCS 8. (a), 22.0 °C and 39%; (b), 22.0 °C and 39%; (c), 22.0 °C and 39%; Figure S8: Stability assessment of the HPTLC method to evaluate stability of sample solutions. Chromatograms obtained from fresh and 8 h-room temperature-stored sample solution, standard mix, and UHM under UV 366 nm; and White light. Track 01, CAS 5; Track 02, Celosin J and Sucrose (standard mix, as increasing *R*_F_); Track 03, UHM (system suitability test reference); Track 04, Celosin I and Celosin H (standard mix, as increasing *R*_F_); Track 05, CCS 8. (a), 0 h, 22.0 °C and 39%; (b), 8 h, 22.0 °C and 39%; Figure S9: Stability assessment of the HPTLC method to evaluate stability of different photographing time based on coloration differences observed. Chromatograms obtained from prepared sample solutions, standard mixed and UHM under UV 366 nm and White light at different time. Track 01, CAS 5; Track 02, Celosin J and Sucrose (standard mix, as increasing *R*_F_); Track 03, UHM (system suitability test reference); Track 04, Celosin I and Celosin H (standard mix, as increasing *R*_F_); Track 05, CCS 8. (a), 0 min; (b), 10 min; (c), 30 min; (d), 60 min; 22.0 °C and 39%; Figure S10: Robustness assessment of the HPTLC method using different developing distance. Chromatograms obtained from prepared sample solutions, standard mix, and UHM under UV 366 nm and White light. Track 01, CAS 5; Track 02, Celosin J and Sucrose (standard mix, as increasing *R*_F_); Track 03, UHM (system suitability test reference); Track 04, Celosin I and Celosin H (standard mix, as increasing *R*_F_); Track 05, CCS 8. (a), 68 mm, 22.0 °C and 39%; (b), 78 mm, 22.0 °C and 39%; (c), 85 mm, 22.0 °C and 39%. Figure S11. Representative HPTLC fingerprints and mass spectra of the spots. (a) linoleic acid in CAS and CCS, detected in positive mode; (b) undefined compound of celosin-type compounds, detected in negative mode.

## Abbreviations

The following abbreviations are used in this manuscript:

ADC	Automatic Developing Chamber
CAS	Celosiae Argentea Semen
CCS	Celosiae Cristatae Semen
ChP 2020	Pharmacopoeia of the People’s Republic of China
ESI	Electrospray Ionization
HPLC	High-Performance Liquid Chromatography
HPTLC	High-Performance Thin-Layer Chromatography
HPTLC-MS	High-Performance Thin-Layer Chromatography coupled with Mass Spectrometry
KHP	Korean Herbal Pharmacopoeia
MS	Mass Spectrometry
PVDF	Polyvinylidene fluoride
SD	Standard deviation
SRAP	Sequence-Related Amplified Polymorphism
SST	System Suitability Test
TLC	Thin-Layer Chromatography
TOF	Time of Flight
