# Supplementary material for: Development of an HPTLC-MS Method for the Differentiation of Celosiae Semen: Celosia argentea Versus C. cristata
Source: Molecules. 2025 Jun 28;30(13):2786. doi: 10.3390/molecules30132786 (PMC12250623; doi:10.3390/molecules30132786)
Supplement: Supplementary file 1 [file molecules-30-02786-s001.zip › molecules-3694957-supplementary.pdf]

Supplementary Material

Development of an HPTLC-MS Method for the Differentiation of Celosiae Semen: *Celosia argentea* versus *C. cristata*

Kyu Won Kim [1,2,†], Geonha Park [2,3,†], Sejin Ku [1,2] and Young Pyo Jang [1-4,*]

[1] Department of Biomedical and Pharmaceutical Sciences, Graduate School, Kyung Hee University, Seoul 02447, Republic of Korea; rbdnjs1127@naver.com (K.-W.K.), zbxl0910@naver.com (S.K.)
[2] Division of Pharmacognosy, College of Pharmacy, Kyung Hee University, Seoul 02447, Republic of Korea
[3] Department of Oriental Pharmaceutical Sciences, College of Pharmacy, Kyung Hee University, Seoul 02447, Republic of Korea; ginapark0326@khu.ac.kr (G.P.)
[3] Department of Integrated Drug Development and Natural Products, Graduate School, Kyung Hee University, Seoul 02447, Republic of Korea; ypjang@khu.ac.kr (Y.-P.J.)

* Correspondence: ypjang@khu.ac.kr; Tel.: +82-2-961-9421
† These authors have equally contributed; Kyu Won Kim and Geonha Park

Table S1. Comparison of mean vertical projection areas of CAS and CCS.

**Celosiae Argentea Semen (CAS)**			**Celosiae Cristatae Semen (CCS)**		
Sample No.	Avg. Area (mm^2)	SD	Sample No.	Avg. Area (mm^2)	SD
1	69.61	6.72	8	171.32	25.31
2	67.90	6.21	9	172.56	33.53
3	70.22	8.77	10	185.83	21.17
4	71.72	8.65	11	159.44	19.15
5	73.77	11.42	12	150.38	19.55
6	74.87	7.72	13	172.55	21.42
7	69.73	8.72	14	158.18	15.63
Total Avg. Area (mm^2)		71.12	Total Avg. Area (mm^2)		167.18
Total SD		2.48	Total SD		11.87

Table S2. Summary of R_F values and variability for system suitability test (SST) acceptance criteria (n=9).

**Substance**	**Mean R_F**	**SD**	**Acceptance Range (Mean±SD)**
[a]	0.844	0.010	0.834 – 0.854
[b]	0.792	0.010	0.782 – 0.802
[c]	0.668	0.015	0.653 – 0.683

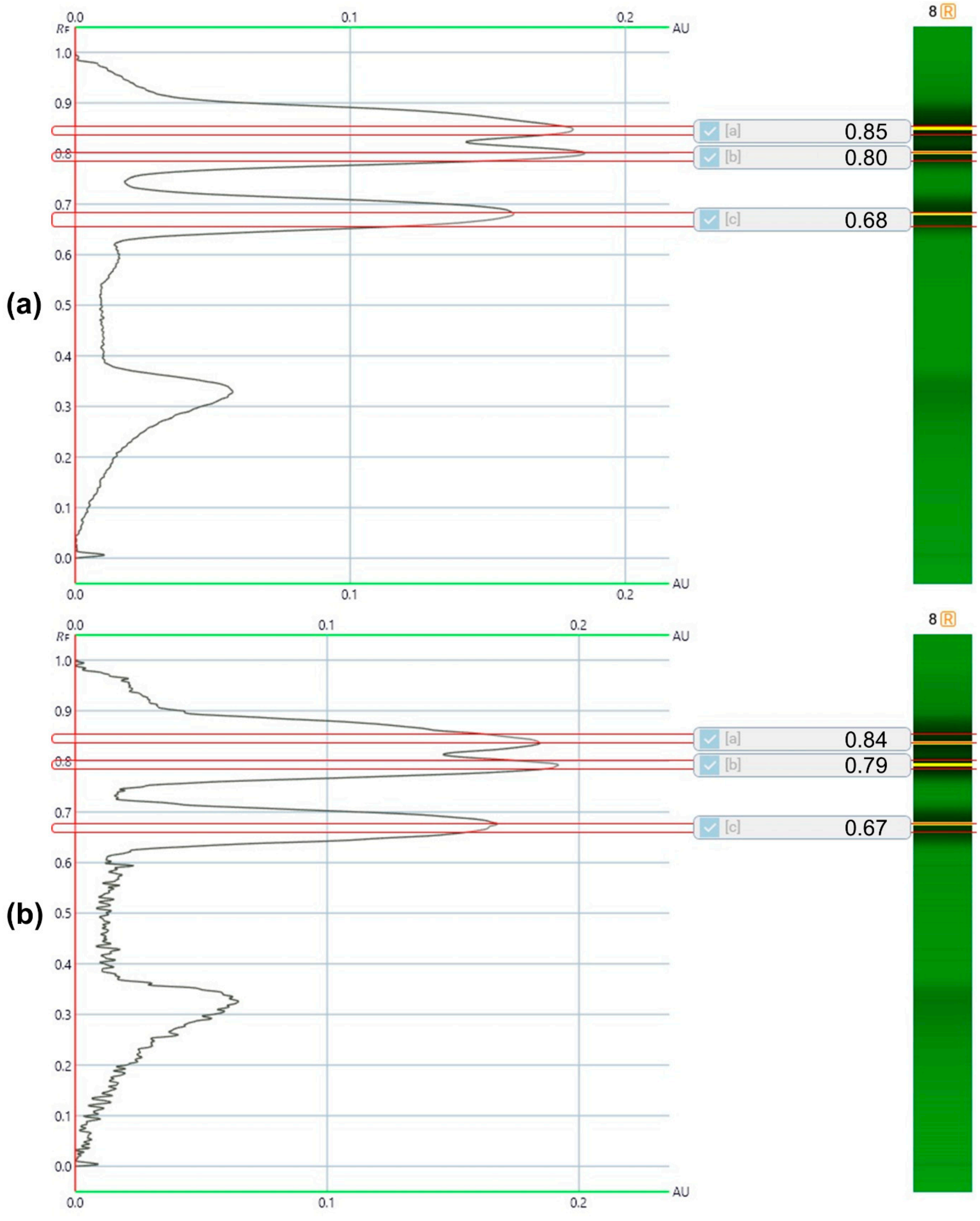

Figure S1. System suitability evaluation of plate 1 (a) and 2 (b) based on acceptance criteria defined using the UHM. The red lines indicate the upper and lower thresholds.

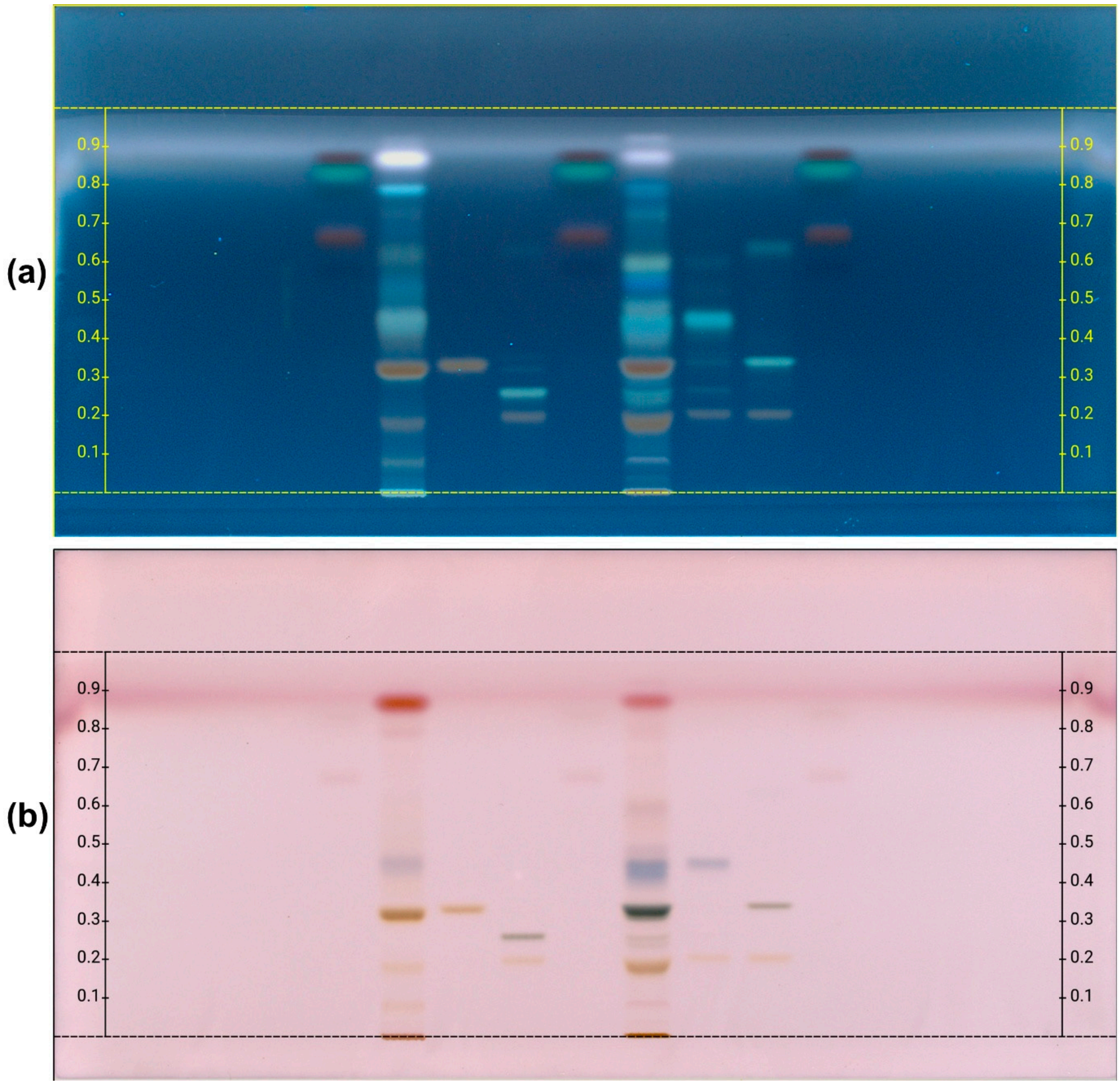

Figure S2. Original image of HPTLC plate 0 under UV (a) 366 nm with an exposure time of 5.034 s and (b) White light with an exposure time of 0.088 s. Track 01 to 03, and 13 to 15, blank; Track 04, 08, and 12, UHM (system suitability test reference); Track 05, CAS 5 (sample); Track 06, Sucrose (standard); Track 06, Celosin J (standard); Track 09, CCS 8 (sample); Tracks 10, Celosin H (standard); Track 11, Celosin I (standard). 20.0°C and 38%.

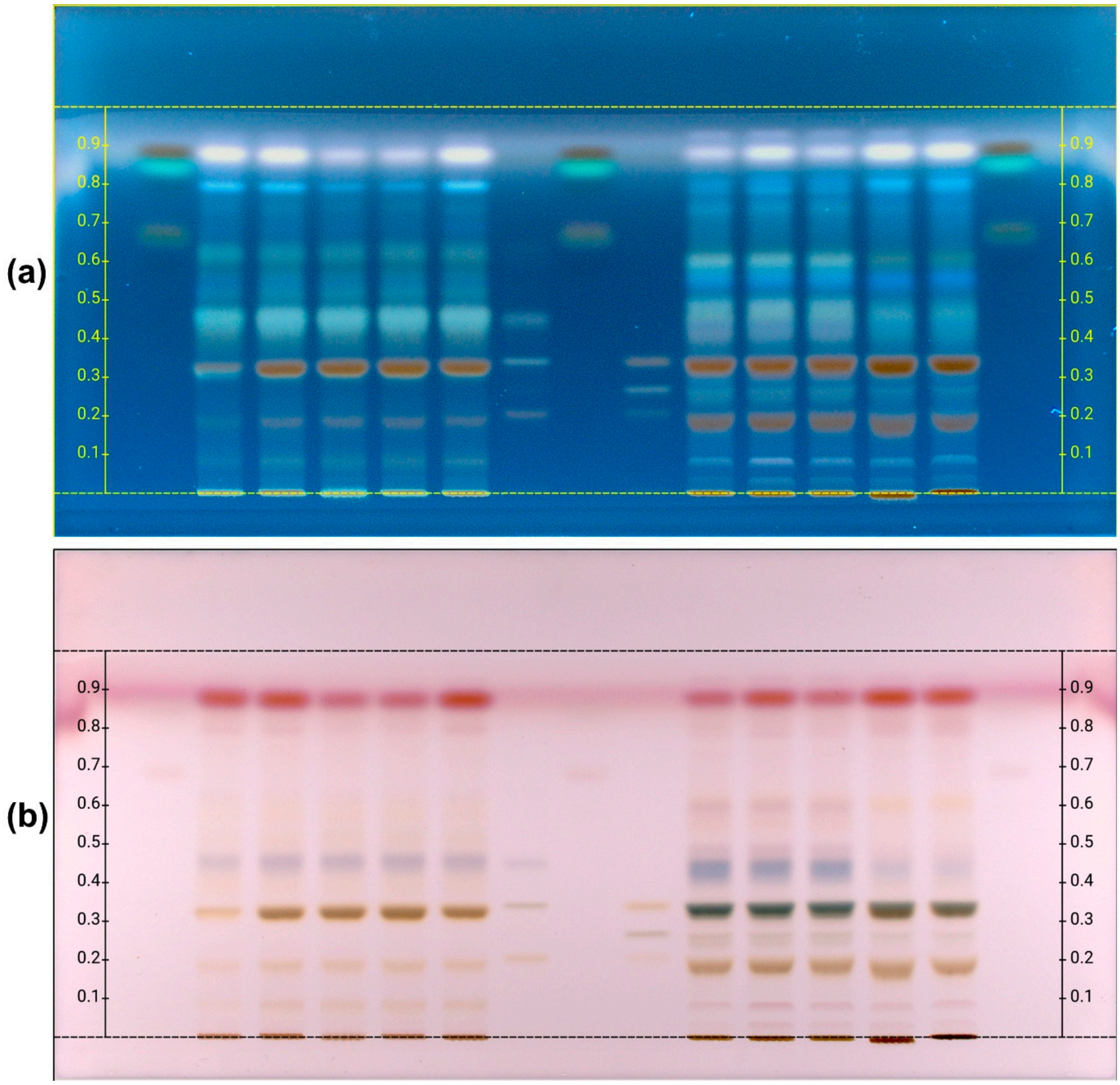

Figure S3. Original image of HPTLC plate 1 under UV (a) 366 nm with an exposure time of 7.165 s and (b) White light with an exposure time of 0.068 s. Track 01, 08, and 15, UHM (system suitability test reference); Track 02 to 06, CAS 1 to CAS 5 (samples); Track 07, Celosin I and Celosin H (standard mix, as increasing R_F); Track 09, Celosin J and Sucrose (standard mix, as increasing R_F); Track 10 to 14, CCS 8 to CCS 12 (samples). 21.0°C and 39%.

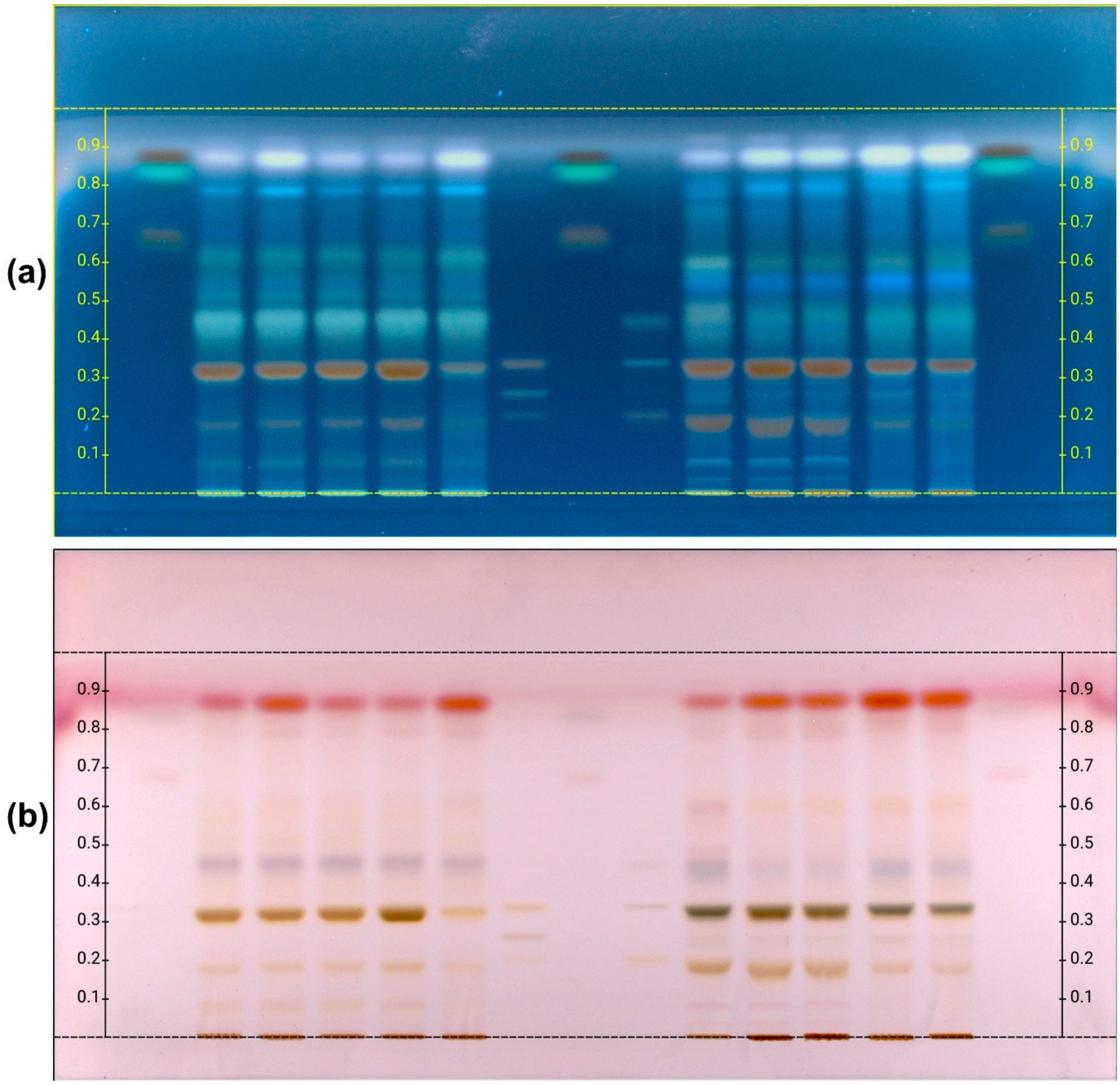

Figure S4. Original image of HPTLC plate 2 under UV (a) 366 nm with an exposure time of 5.729 s and (b) White light with an exposure time of 0.056 s. Track 01, 08, and 15, UHM (system suitability test reference); Track 02 to 04, CAS 3, CAS 2, and CAS 4 (samples); Track 05 and 06, CAS 6 and CAS 7 (samples); Track 07, Celosin J and Sucrose (standard mix, as increasing R_F); Track 09, Celosin I and Celosin H (standard mix, as increasing R_F); Track 10 to 14, CCS 10 to CCS 14 (samples). 21.0°C and 39%.

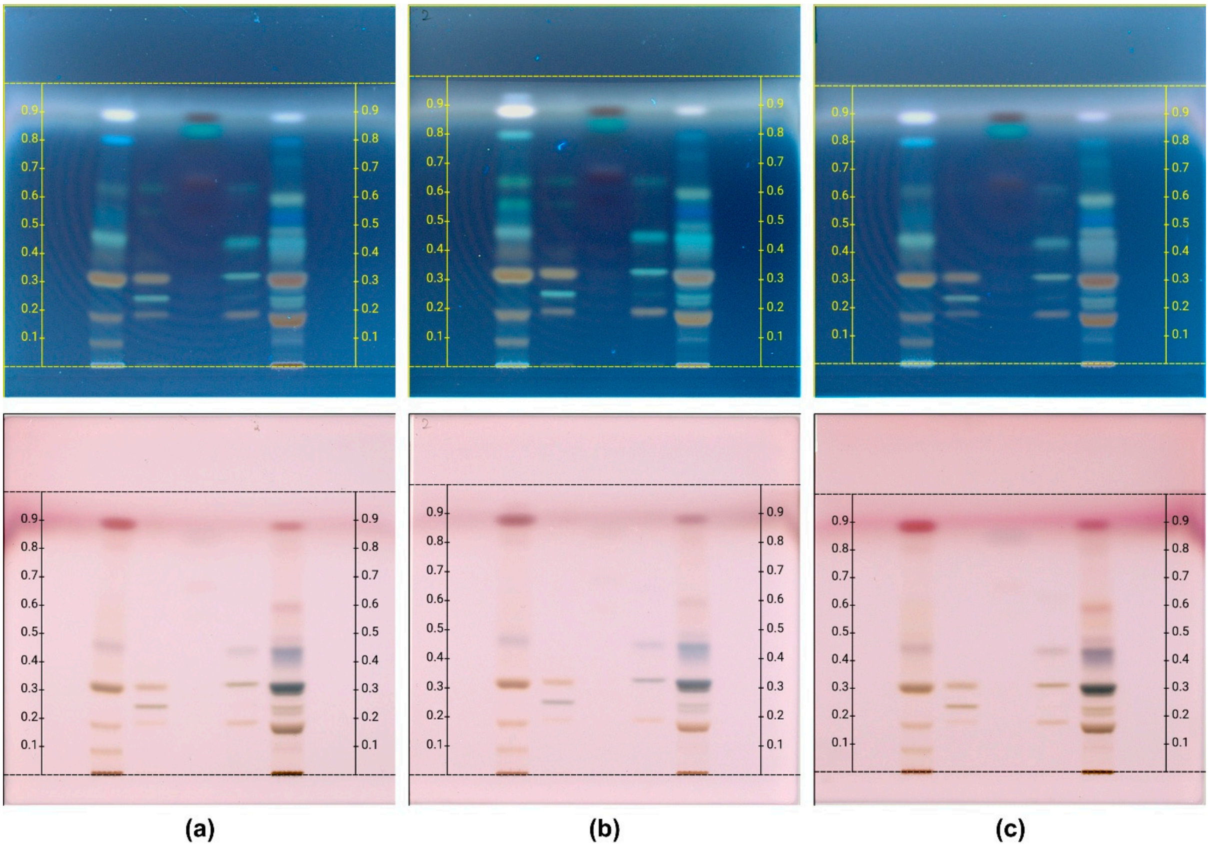

Figure S5. Repeatability assessment of the HPTLC method to evaluate intra-day precision based on three replicate analyses. Chromatograms obtained from three independently prepared sample solutions of the same material, standard mix, and UHM under UV 366 nm and White light. Track 01, CAS 5; Track 02, Celosin J and Sucrose (standard mix, as increasing R_F); Track 03, UHM (system suitability test reference); Track 04, Celosin I and Celosin H (standard mix, as increasing R_F); Track 05, CCS 8. (a), 21.0°C and 39%; (b), 21.0°C and 39%; (c), 22.0°C and 39%.

Table S3. R_F Values of Celosin J, Sucrose, Celosin I, and Celosin H in the intra-day precision of the Repeatability assessment. The identifiers (a), (b), and (c) correspond to the respective plates illustrated in Figure S5.

Compound	Sample	(a)	(b)	(c)	Average	SD	Total average	Total SD
Celosin J	Standard	0.24	0.25	0.24	0.243	0.006	0.240	0.006
	CCS	0.24	0.24	0.23	0.237	0.006		
Sucrose	Standard	0.31	0.32	0.31	0.313	0.006	0.311	0.006
	CAS	0.31	0.32	0.31	0.313	0.006		
	CCS	0.31	0.31	0.30	0.307	0.006		
Celosin I	Standard	0.32	0.32	0.31	0.317	0.006	0.317	0.005
	CCS	0.32	0.32	0.31	0.317	0.006		
Celosin H	Standard	0.44	0.45	0.44	0.443	0.006	0.442	0.004
	CCS	0.44	0.44	0.44	0.440	0.000		

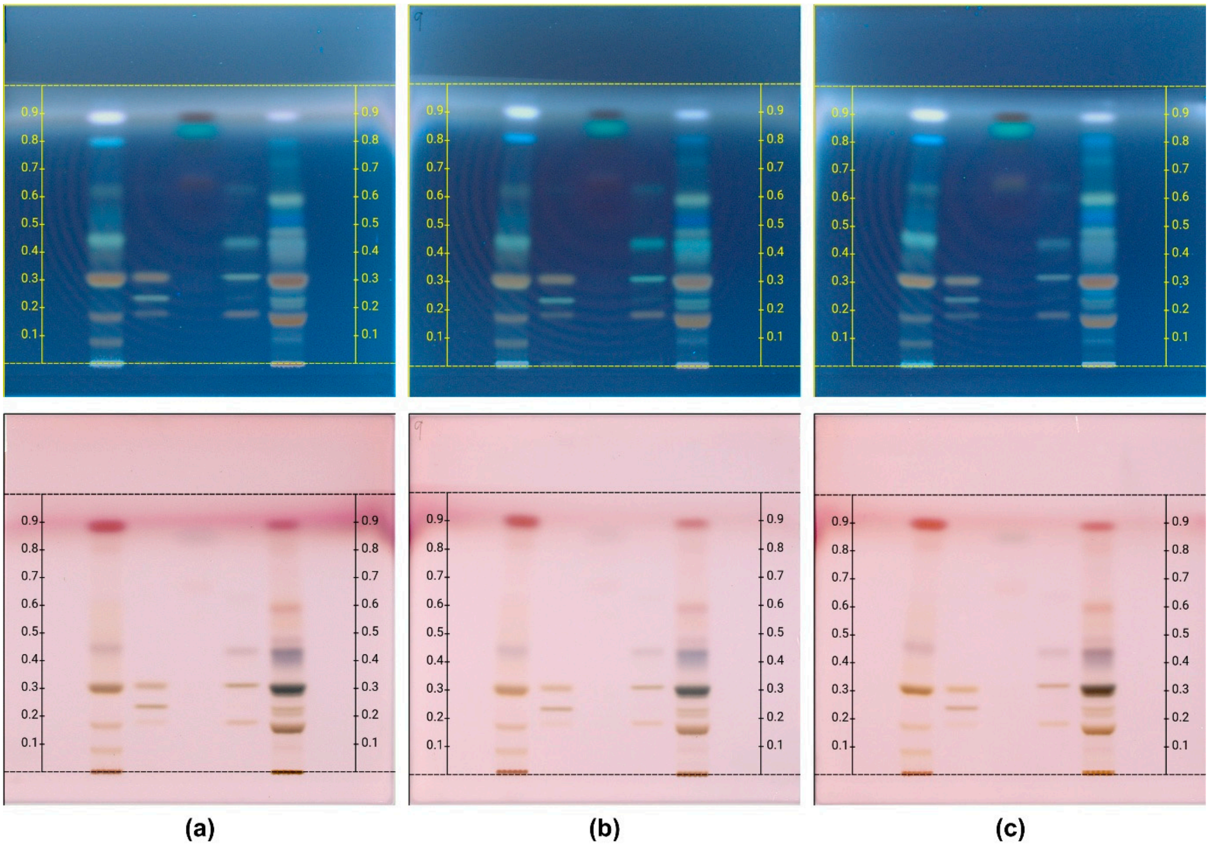

Figure S6. Repeatability assessment of the HPTLC method to evaluate inter-day precision based on analyses conducted on three separate days. Chromatograms obtained from three different sample solutions prepared on three days, standard mix, and UHM under UV 366 nm and White light. Track 01, CAS 5; Track 02, Celosin J and Sucrose (standard mix, as increasing R_F); Track 03, UHM (system suitability test reference); Track 04, Celosin I and Celosin H (standard mix, as increasing R_F); Track 05, CCS 8. (a), 22.0°C and 39%; (b), 22.0°C and 39%; (c), 20.0°C and 39%.

Table S4. R_F Values of Celosin J, Sucrose, Celosin I, and Celosin H in the inter-day precision of the Repeatability assessment. The identifiers (a), (b), and (c) correspond to the respective plates illustrated in Figure S6.

Compound	Sample	(a)	(b)	(c)	Average	SD	Total average	Total SD
Celosin J	Standard	0.24	0.24	0.24	0.240	0.000	0.237	0.005
	CCS	0.23	0.23	0.24	0.233	0.006		
Sucrose	Standard	0.31	0.31	0.31	0.310	0.000	0.308	0.004
	CAS	0.31	0.31	0.31	0.310	0.000		
	CCS	0.30	0.30	0.31	0.303	0.006		
Celosin I	Standard	0.31	0.31	0.32	0.313	0.006	0.313	0.005
	CCS	0.31	0.31	0.32	0.313	0.006		
Celosin H	Standard	0.44	0.44	0.44	0.440	0.000	0.438	0.004
	CCS	0.44	0.43	0.44	0.437	0.006		

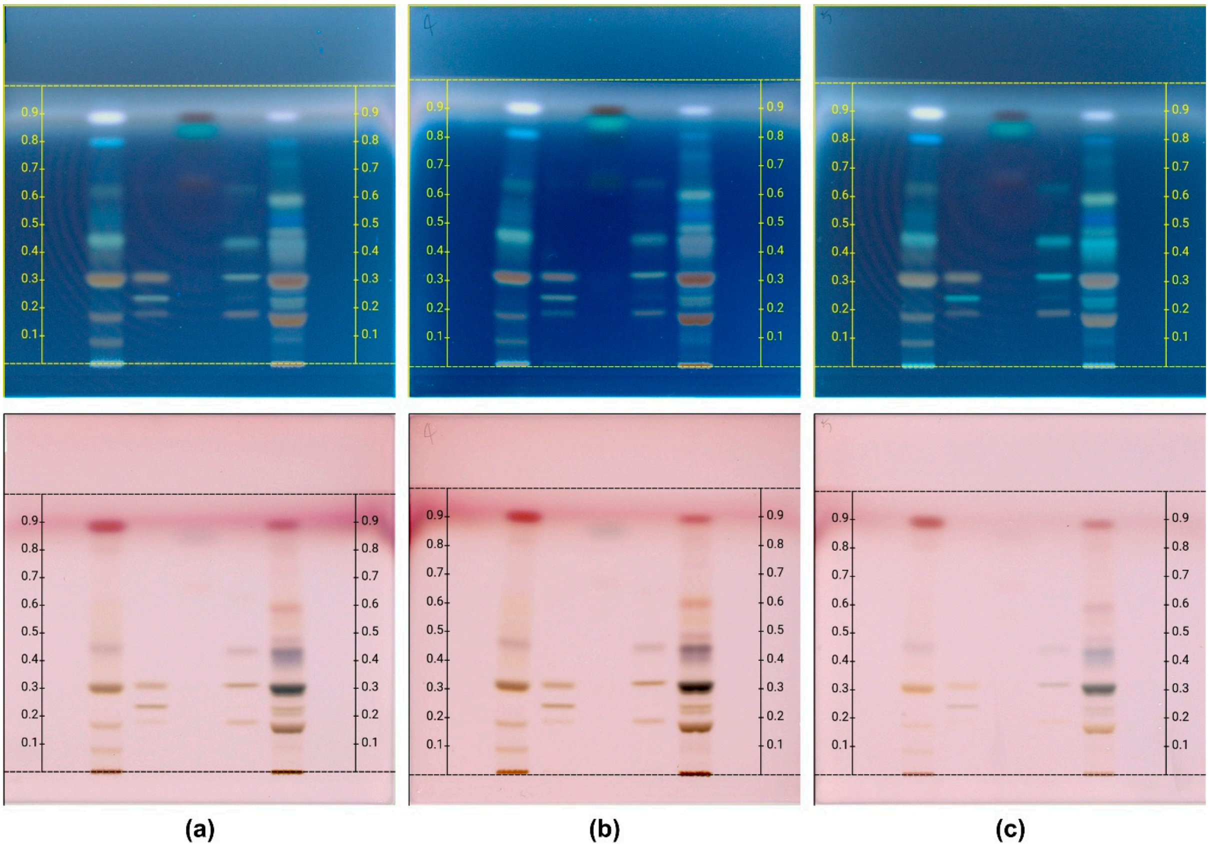

Figure S7. Repeatability assessment of the HPTLC method to evaluate inter-analyst precision based on analysis of different analysts. Chromatograms obtained from three sample solutions independently prepared by 3 operators, standard mix, and UHM under UV 366 nm and White light. Track 01, CAS 5; Track 02, Celosin J and Sucrose (standard mix, as increasing R_F); Track 03, UHM (system suitability test reference); Track 04, Celosin I and Celosin H (standard mix, as increasing R_F); Track 05, CCS 8. (a), 22.0°C and 39%; (b), 22.0°C and 39%; (c), 22.0°C and 39%.

Table S5. R_F Values of Celosin J, Sucrose, Celosin I, and Celosin H in the inter-analyst precision of the Repeatability assessment. The identifiers (a), (b), and (c) correspond to the respective plates illustrated in Figure S7.

Compound	Sample	(a)	(b)	(c)	Average	SD	Total average	Total SD
Celosin J	Standard	0.24	0.24	0.24	0.240	0.000	0.238	0.004
	CCS	0.23	0.24	0.24	0.237	0.006		
Sucrose	Standard	0.31	0.31	0.32	0.313	0.006	0.310	0.005
	CAS	0.31	0.31	0.31	0.310	0.000		
	CCS	0.30	0.31	0.31	0.307	0.006		
Celosin I	Standard	0.31	0.32	0.32	0.317	0.006	0.313	0.005
	CCS	0.31	0.31	0.31	0.310	0.000		
Celosin H	Standard	0.44	0.44	0.44	0.440	0.000	0.440	0.000
	CCS	0.44	0.44	0.44	0.440	0.000		

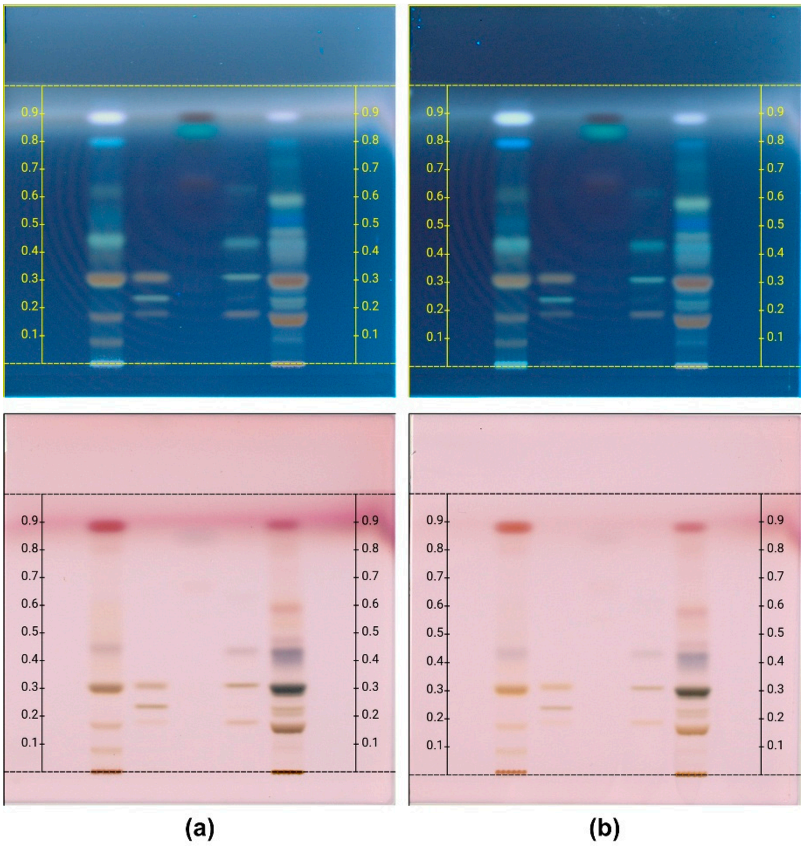

Figure S8. Stability assessment of the HPTLC method to evaluate stability of sample solutions. Chromatograms obtained from fresh and 8 hours-room temperature-stored sample solution, standard mix, and UHM under UV 366 nm; and White light. Track 01, CAS 5; Track 02, Celosin J and Sucrose (standard mix, as increasing R_F); Track 03, UHM (system suitability test reference); Track 04, Celosin I and Celosin H (standard mix, as increasing R_F); Track 05, CCS 8. (a), 0 hour, 22.0°C and 39%; (b), 8 hours, 22.0°C and 39%.

Table S6. R_F Values of Celosin J, Sucrose, Celosin I, and Celosin H in the stability of sample solutions of the Stability assessment. The identifiers (a) and (b) correspond to the respective plates illustrated in Figure S8.

Compound	Sample	(a)	(b)	Average	SD	Total average	Total SD
Celosin J	Standard	0.24	0.24	0.240	0.000	0.235	0.006
	CCS	0.23	0.23	0.230	0.000		
Sucrose	Standard	0.31	0.31	0.310	0.000	0.307	0.005
	CAS	0.31	0.31	0.310	0.000		
	CCS	0.30	0.30	0.300	0.000		
Celosin I	Standard	0.31	0.31	0.310	0.000	0.308	0.005
	CCS	0.31	0.30	0.305	0.007		
Celosin H	Standard	0.44	0.43	0.435	0.007	0.435	0.006
	CCS	0.44	0.43	0.435	0.007		

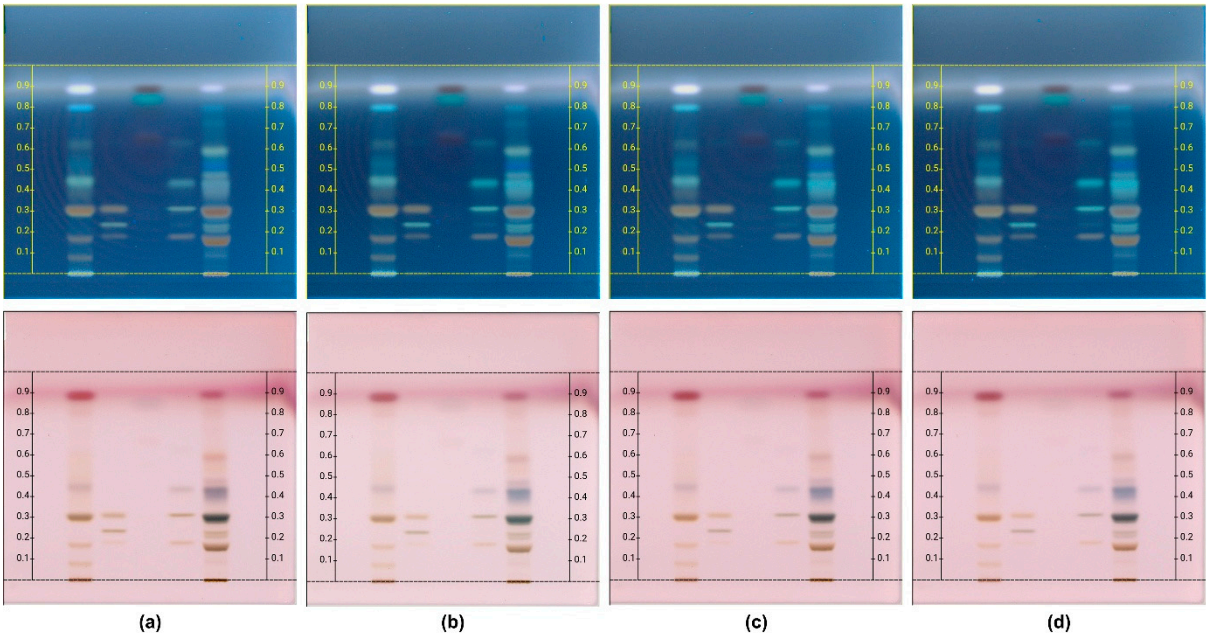

Figure S9. Stability assessment of the HPTLC method to evaluate stability of different photographing time based on coloration differences observed. Chromatograms obtained from prepared sample solutions, standard mixed and UHM under UV 366 nm and White light at different time. Track 01, CAS 5; Track 02, Celosin J and Sucrose (standard mix, as increasing R_F); Track 03, UHM (system suitability test reference); Track 04, Celosin I and Celosin H (standard mix, as increasing R_F); Track 05, CCS 8. (a), 0 min; (b), 10 min; (c), 30 min; (d), 60 min; 22.0°C and 39%.

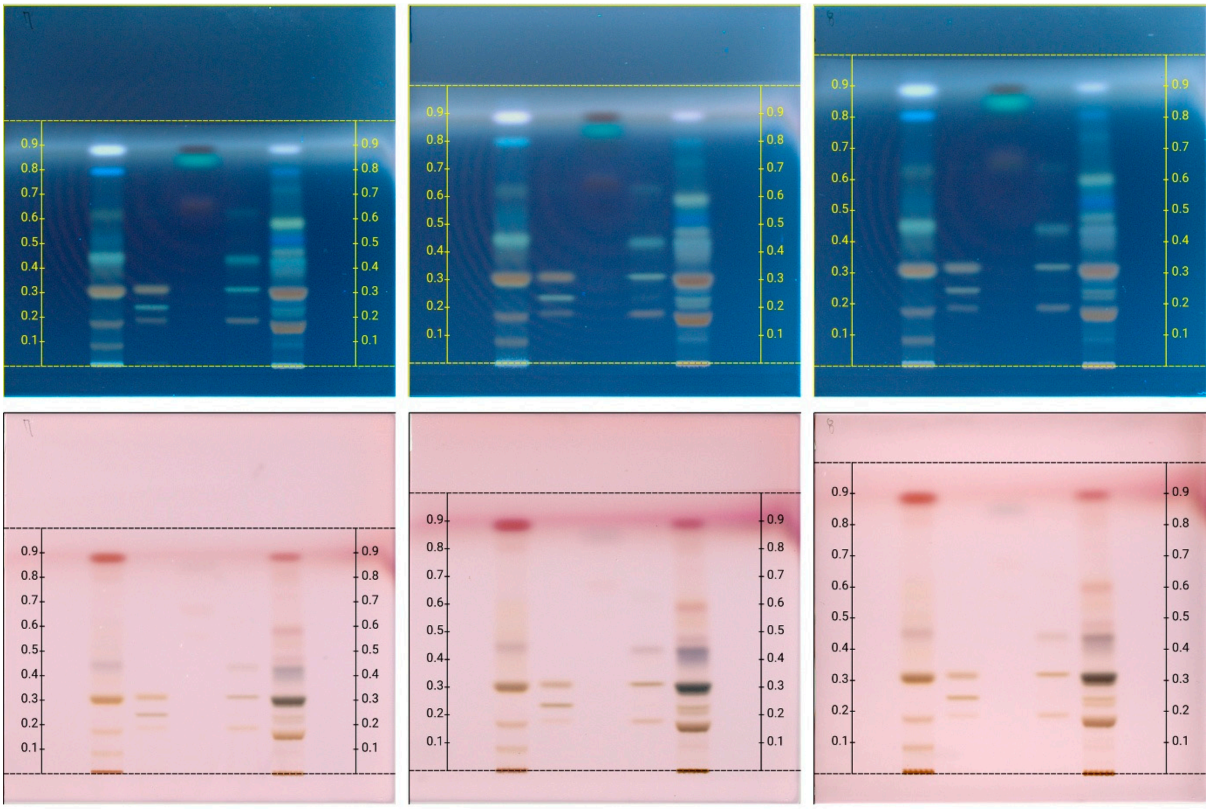

Figure S10. Robustness assessment of the HPTLC method using different developing distance. Chromatograms obtained from prepared sample solutions, standard mix, and UHM under UV 366 nm and White light. Track 01, CAS 5; Track 02, Celosin J and Sucrose (standard mix, as increasing R_F); Track 03, UHM (system suitability test reference); Track 04, Celosin I and Celosin H (standard mix, as increasing R_F); Track 05, CCS 8. (a), 68 mm, 22.0°C and 39%; (b), 78 mm, 22.0°C and 39%; (c), 85 mm, 22.0°C and 39%.

Table S7. R_F Values of Celosin J, Sucrose, Celosin I, and Celosin H in the Robustness assessment using different developing distance. The identifiers (a), (b), and (c) correspond to the respective plates illustrated in Figure S10.

Compound	Sample	(a)	(b)	(c)	Average	SD	Total average	Total SD
Celosin J	Standard	0.24	0.24	0.24	0.240	0.000	0.237	0.005
	CCS	0.23	0.23	0.24	0.233	0.006		
Sucrose	Standard	0.31	0.31	0.32	0.313	0.006	0.309	0.006
	CAS	0.31	0.31	0.31	0.310	0.000		
	CCS	0.30	0.30	0.31	0.303	0.006		
Celosin I	Standard	0.31	0.31	0.32	0.313	0.006	0.313	0.005
	CCS	0.31	0.31	0.32	0.313	0.006		
Celosin H	Standard	0.44	0.44	0.44	0.440	0.000	0.440	0.000
	CCS	0.44	0.44	0.44	0.440	0.000		

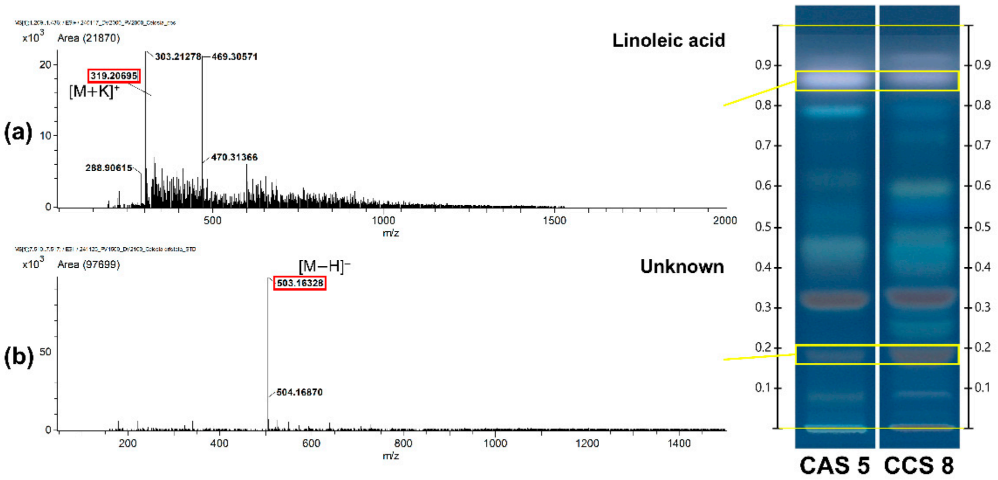

Figure S11. Representative HPTLC fingerprints and mass spectra of the spots. (a) linoleic acid in CAS and CCS, detected in positive mode; (b) undefined compound of celosin-type compounds, detected in negative mode.
